# Follistatin is a crucial chemoattractant for mouse decidualized endometrial stromal cell migration by JNK signalling

**DOI:** 10.1111/jcmm.17648

**Published:** 2022-12-18

**Authors:** Guole Liu, Yan Qi, Jiandong Wu, Francis Lin, Zhonghui Liu, Xueling Cui

**Affiliations:** ^1^ Department of Immunology, College of Basic Medical Sciences Jilin University Changchun China; ^2^ Institute of Biomedical and Health Engineering, Shenzhen Institute of Advanced Technology, Chinese Academy of Sciences Shenzhen China; ^3^ Department of Physics and Astronomy University of Manitoba Winnipeg Manitoba Canada; ^4^ Department of Genetics, College of Basic Medical Sciences Jilin University Changchun China

**Keywords:** activin a, endometrial stromal cells, Follistatin, microfluidic device, migration

## Abstract

Follistatin (FST) and activin A as gonadal proteins exhibit opposite effects on follicle‐stimulating hormone (FSH) release from pituitary gland, and activin A‐FST system is involved in regulation of decidualization in reproductive biology. However, the roles of FST and activin A in migration of decidualized endometrial stromal cells are not well characterized. In this study, transwell chambers and microfluidic devices were used to assess the effects of FST and activin A on migration of decidualized mouse endometrial stromal cells (d‐MESCs). We found that compared with activin A, FST exerted more significant effects on adhesion, wound healing and migration of d‐MESCs. Similar results were also seen in the primary cultured decidual stromal cells (DSCs) from uterus of pregnant mouse. Simultaneously, the results revealed that FST increased calcium influx and upregulated the expression levels of the migration‐related proteins MMP9 and Ezrin in d‐MESCs. In addition, FST increased the level of phosphorylation of JNK in d‐MESCs, and JNK inhibitor AS601245 significantly attenuated FST action on inducing migration of d‐MESCs. These data suggest that FST, not activin A in activin A‐FST system, is a crucial chemoattractant for migration of d‐MESCs by JNK signalling to facilitate the successful uterine decidualization and tissue remodelling during pregnancy.

## 1 INTRODUCTION

Pregnancy is a complex physiological process, including the interaction between foetus and mother to maintain foetal development. Decidualization is a tissue remodelling process involving a variety of cell types in maternal uterus during pregnancy. The transformation of endometrial stromal cells (ESCs) into specialized secretory decidual cells is a key step in embryo implantation and survival. During decidualization, spindle‐shaped fibroblast‐like ESCs undergo dramatic morphological changes and differentiate into cobblestone‐shaped decidual cells.^1^ The blastocyst contacts the uterine epithelial cells, accompanied by the proliferation and differentiation of stromal cells to prepare for embryo implantation.^2^ The decidualization process of mouse uterus occurs on Day 4.5 postcoitum (Day 0.5‐vaginal plug), after that, a receptive endometrium is created for embryo implantation. The unique decidual environment can provide nutrition and growth factors for implanted embryos, regulate the invasion of trophoblast cells to decidua, control the activity of immune cells at the maternal foetal interface and establish immune tolerance.^3^, ^4^ The migration of decidualized ESCs is a critical event during decidualization, accompanying with the invasion of extravillous trophoblast cells into the decidual tissue,^5^ but the systematic research on the mechanism of migration of the decidualized ESCs is still limited.

Activin is the member of transforming growth factor‐beta superfamily, and a double‐chain glycoprotein connected by disulfide β subunit composition. It is isolated and identified from follicular fluid of ovary, and is so named because it can promote the secretion of follicle‐stimulating hormone (FSH) by pituitary. According to the type of β subunit, activins are divided into activin A (βAβA), activin AB (βAβB) and activin B (βBβB).^6^ Among them, activin A (Act A) is widely distributed in various tissues and well‐studied. It serves as a sexual hormone regulatory protein that participates in a variety of physiological and pathological processes including regulation of inflammation, fibrosis, tumorigenesis, neurotransmission, angiogenesis and embryogenesis.^7^, ^8^, ^9^ There are two signalling pathways of activin A, canonical SMADs‐dependent signalling pathway and non‐canonical signalling pathway. In the canonical signalling pathway, activin A binds to activin type II receptor (ActRII) to recruit and activate activin type I receptor (ActRI). The serine/threonine kinase residues of ActRI are phosphorylated, which then induce phosphorylation of SMAD2 and SMAD3. Moreover, the SMAD2/3/4 complex is formed to promote gene expression.^10^, ^11^, ^12^ In reproductive biology, activin A plays an important role in the regulation of hormone secretion, menstrual cycle and decidualization.^13^ Previous studies have reported that the expression of activin A receptor in ESCs increases in early pregnancy,^14^, ^15^ and activin A can promote decidualization of human ESCs,^16^ while activin A deficient mice develop to term but die within 24 h of birth.^17^ The physiological role of activin A derived from endometrium is unclear, but in the paracrine process, activin A can regulate cell differentiation, promote cell proliferation and participate in tissue remodelling and inflammatory response. These physiological processes are consistent with decidualization events.^18^, ^19^, ^20^

As activin binding protein, follistatin (FST) is a single chain glycoprotein that is also isolated and identified from follicular fluid of ovary. Contrary to activin A, it can inhibit the secretion of FSH by pituitary. FST has a high affinity for activin A, which can prevent activin A from binding to its receptor and neutralize its biological effect.^21^ FST also plays an important role in regulating activin bioavailability in circulation and within tissues.^22^ In reproductive biology, studies have reported that both endometrial epithelial cells and decidual stromal cells can secrete FST.^14^ Conditional knockout of FST impairs the receptivity and decidualization of mouse endometrium, resulting in serious reproductive defects.^23^ These studies suggest that FST and activin A play an important role in decidualization of uterus during pregnancy.

Activin A and FST are essential hormone regulatory proteins during decidualization, but their effects on the decidual cell migration remain to be clarified. Therefore, this study analysed the effects of activin A and FST on the migration of decidualized mouse endometrial stromal cells (d‐MESCs) in vitro and primary cultured decidual stromal cells (DSCs) from uterus of pregnant mouse. The results revealed that compared to activin A, FST is a more effective chemoattractant for inducing migration of d‐MESCs, which may provide a new insight for reducing the abortion rate caused by decidual dysplasia and facilitating successful embryo implantation and development.

## 2 MATERIALS AND METHODS

### 2.1 Reagents

Recombinant activin A and FST were purchased from R&D systems (Minneapolis, Mn, United States). DNase I and Liberase™ Research Grade were obtained from Roche (Mannheim, Germany). Progesterone (P_4_) and Estradiol (E_2_) were provided by TCI (Shanghai, China). Giemsa cytological stain was purchased from Sigma Aldrich (Oakville, ON, Canada). Fluo‐4 was from Thermo Fisher Scientific (Ottawa, ON, Canada). The antibodies against GAPDH (abs132004), MMP2 (abs130432) and MMP9 (abs155182) were obtained from Absin (Shanghai, China). The antibodies against Ezrin (3145S), Vimentin (5741S), p‐ERK1/2 (4370S), ERK1/2 (4695S), p‐SMAD3 (9520S) and SMAD3 (9523S) were purchased from Cell Signaling Technology (Danvers, MA, United States). The antibodies against p‐p38 (WLP1576), p38 (WL00764), p‐JNK (WL01813) and JNK (WL01295) were bought from Wanlei Biotechnology (Shenyang, China). JNK inhibitor AS601245 was obtained from Absin (Shanghai, China).

### 2.2 Primary ESCs/DSCs isolation and culture

The endometrial stromal cells (ESCs) were isolated as previously described with slight modification.^24^ Briefly, the mouse endometrium was separated under a dissecting microscope and cut into small pieces, then digested in media contained Liberase (0.125 mg/ml), DNase (2 mg/ml) and 0.25% trypsin for 1 h on ice followed by 1 h at room temperature, and 10 min at 37°C in a shaking water bath. Later, discard the supernatant and digest the remaining tissues in DMEM/F12 containing Liberase (0.125 mg/ml) and DNase (2 mg/ml) at 37°C for 40 min. Finally, a 70‐μm filter was used to remove the undigested tissue pieces, and the cells were collected by centrifugation at 1000 rpm for 4 min and washed twice with PBS. Decidual stromal cells (DSCs) were isolated from uterus of pregnant mouse by using the same method.

The isolated ESCs or DSCs were cultured in DMEM/F12 with 10% foetal bovine serum (FBS, BI, Israel) containing 100 U/ml Penicillin and 0.1 mg/ml Streptomycin (Biosharp, China).

### 2.3 In vitro ESCs decidualization

In vitro decidualized mouse ESCs were carried out as previously described.^25^, ^26^, ^27^ Briefly, mouse ESCs were cultured in 2% FBS‐DMEM/F12 containing 10 nM E_2_ and 1 μM P_4_. After 5 days treatment, the d‐MESCs were harvested for subsequent experiments.

### 2.4 Immunocytochemical staining

Identification of the isolated mouse ESCs and DSCs was performed by immunocytochemical staining. In brief, mouse ESCs or DSCs were cultured in 2% FBS‐DMEM/F12 and then fixed with 4% paraformaldehyde for 20 min. Immunocytochemical staining was carried out using primary rabbit anti‐Vimentin antibody and second anti‐Rabbit IgG antibody‐Streptomyces Ovalbumin‐Biotin Detection System (SP9001, Beijing Zhongshan Jinqiao Biotechnology). Immunoreactivities were visualized with the 3,3’‐Diaminobenzidine (DAB, ZLI‐9017, Beijing Zhongshan Jinqiao Biotechnology). Haematoxylin (Biosharp) was used to stain nucleus. Anti‐Vimentin immunoreactivities were observed under CKX53 (Olympus) microscope.

### 2.5 RT‐PCR

Total RNA was extracted from d‐MESCs using TRIzol reagent (Takara), and 1 μg of total RNA was used for cDNA synthesis using the PrimeScript Reverse Transcription Kit (Takara). PCR was carried out using PCR Kit (Takara) under the following conditions: 95°C for 90 s, followed by 35 cycles of (94°C for 30 s, 56°C for 30 s, 72°C for 1 min) with a final extension at 72°C for 10 min. PCR products were separated by 2% agarose gel electrophoresis and stained with Super GelRed (US EVERBRIGHT). The cDNA bands were analysed by Image J software (16.0.1), and gene expression levels were normalized against GAPDH. Primer sequences were shown in Table S1.

### 2.6 Cell viability assay

Cell Counting Kit‐8 (CCK‐8) (Biosharp) was used to detect the cell viability. d‐MESCs/DSCs were seeded into a 96‐well plate and incubated in 2% FBS‐DMEM/F12 containing activin A (2.5 ng/ml–20 ng/ml) and FST (5 ng/ml–40 ng/ml) at 37°C in 5% CO_2_ for 24 h. Then, 10 μl of CCK‐8 reagent was added to each well and incubated for another 2 h. The absorbance of 450 nm (A 450 nm) was detected by a microplate spectrophotometer.

### 2.7 Real‐time cell analysis

Cell adhesion was monitored in the xCELLigence Real‐Time Cell Analysis (RTCA; ACEA Biosciences Inc.) system using E‐Plate 16 (ACEA Biosciences Inc.). Briefly, a total 50 μl of 2% FBS‐DMEM/F12 was added into the plates, and baseline measurements were taken. d‐MESCs (1 × 10^4^ cells) were then seeded into the wells in 150 μl of 2% FBS‐DMEM/F12 with or without activin A and/or FST. Cells were monitored every 15 min for 6 h.

### 2.8 Wound healing assay

d‐MESCs/DSCs were seeded into 12‐well plates at a density of 1 × 10^5^ cells per well and incubated at 37°C in 5% CO_2_ to form a sub‐confluent monolayer. Then, a scratch‐wound was produced in the confluent monolayers using a sterile 200 μl pipette tip, and the detached cells were removed with washing. Cells were further cultured in 2% FBS‐DMEM/F12 containing 5 ng/ml activin A and/or 10 ng/ml FST. The images were obtained using an inverted microscope at 0 h, 12 h and 24 h, and the surface area of the scratch at different time points was measured and analysed using Image J software version (16.0.1) (National Institutes of Health, United States).

### 2.9 Transwell chamber assay

Migration of d‐MESCs was examined using transwell chamber assay. Briefly, 3 × 10^4^ cells were seeded in the upper chambers (8 μm pore size; Corning) in 200 μl DMEM/F12 with 2% FBS. The lower compartments of the chambers were filled with 500 μl of 2% FBS‐DMEM/F12 containing activin A (5 ng/ml) or/and FST (10 ng/ml). After 6 h incubation at 37°C, non‐migratory cells on the upper side of the membrane were removed by gently swiping with cotton swabs. The cells on the lower surface of the membrane were fixed with 4% paraformaldehyde for 20 min and then stained with Giemsa. The cell numbers were counted in five randomly chosen fields in each group.

### 2.10 Microfluidic cell migration assay

Microfluidic devices were fabricated using the standard photolithography and soft‐lithography technique as described previously.^28^ d‐MESCs/DSCs (3 × 10^5^/ml) were loaded into the cell inlets and allowed to align in the docking structures. The chemoattractant solutions included 5 ng/ml activin A, 10 ng/ml FST or 5 ng/ml activin A combined with 10 ng/ml FST in 2% FBS‐DMEM/F12. Cell migration images were acquired every 4 h for 12 h/24 h. The migration distance was measured by image J software version (16.0.1). The migration track was analysed by Chemotaxis and Migration Tool (ibidi GmbH). The chemotactic index (C.I.) is defined as the ratio of displacement towards the gradient to the total migration distance of the cell and were calculated.^28^

### 2.11 Western blotting

d‐MESCs were treated with 5 ng/ml activin A and/or 10 ng/ml FST, and then lysed on ice in protein extraction reagent. Then, proteins were quantified using BCA Protein Assay Kit (Thermo Scientific, United States). Whole cell extract proteins (10–30 μg) were electrophoresed in 10% SDS‐PAGE gels and transferred onto polyvinylidene difluoride (PVDF) membranes (Merck Millipore). Bound antibodies were detected with ECL detection reagent (GE Healthcare), and images were captured by Tanon‐4600. The intensity of the target proteins blots was quantified by Image J software version (16.0.1) (National Institutes of Health, United States).

### 2.12 Calcium flux assay

d‐MESCs were resuspended in 2% FBS‐DMEM/F12 with 4 μM Fluo‐4 in dark for 40 min, and cells were recovered for an additional 30 min. After that, the cells were divided into tubes for flow cytometry. Firstly, the cells were collected for 1 min as the baseline (F_0_), then activin A (5 ng/ml) and/or FST (10 ng/ml) in 2% FBS‐DMEM/F12 were added to stimulate d‐MESCs, and the Fluo‐4 signal (F) was recorded for another 3 min. Fluo‐4 signal indicates intracellular calcium level. The kinetics of Fluo‐4 intensity was analysed by Flowjo software (FlowJo LLC. Ashland). The changes of intracellular calcium level were normalized to the baseline for comparison (F/F_0_). ACEA NovoCyte (Agilent) was used to collect the Fluo‐4 signal.

### 2.13 Statistical analysis

All data were shown as means ± SD. Statistical evaluation was conducted using a Student's *t*‐test or one‐way anova followed by Tukey's multiple comparisons test. A significant difference was defined as *p <* 0.05.

## 3 RESULTS

### 3.1 Effects of activin A and FST on viability of d‐MESCs

**FIGURE 1:**
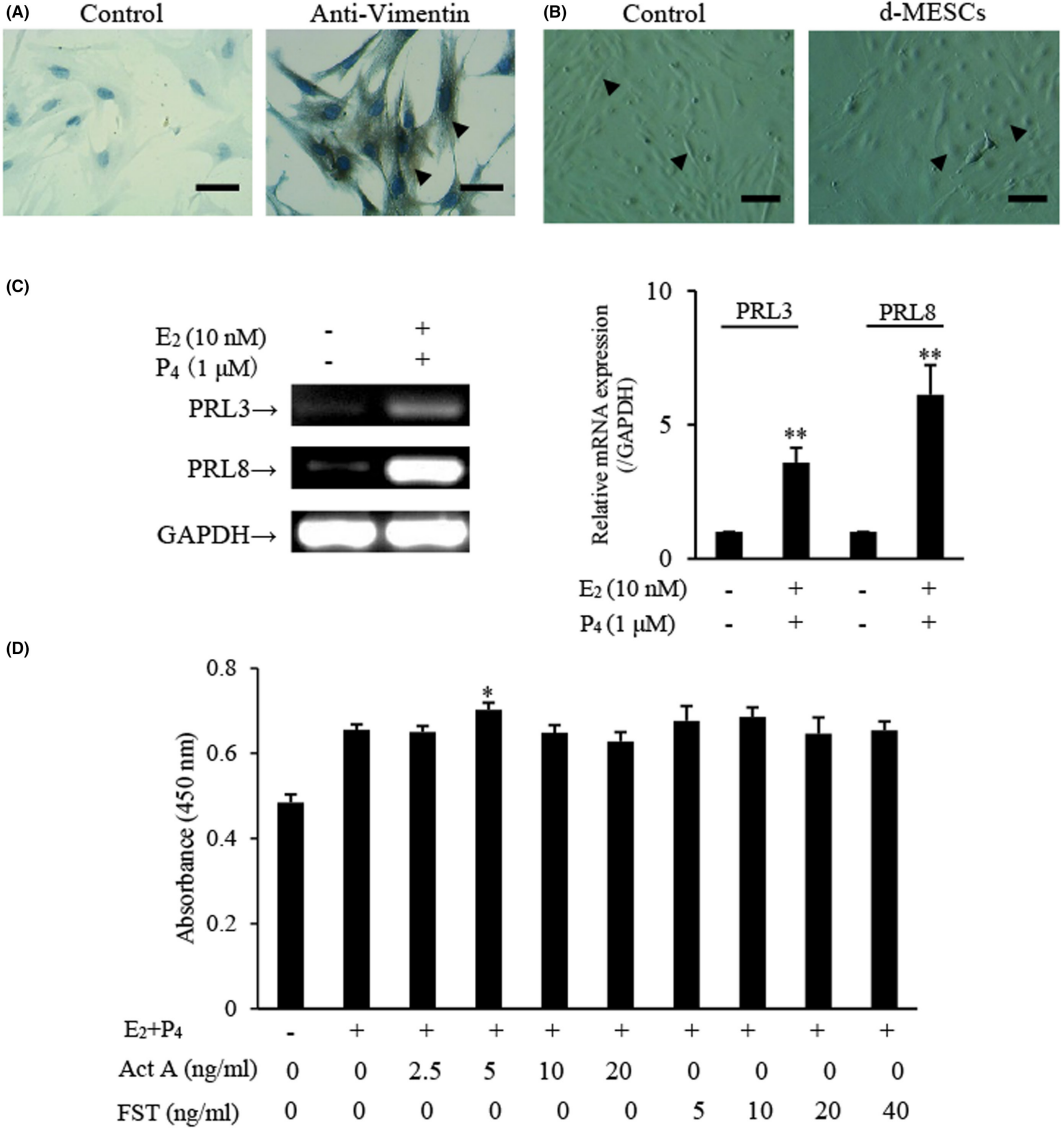
Effects of activin A and FST on viability of d‐MESCs. (A) Immunocytochemical staining was carried out to examine Vimentin expression in isolated mouse ESCs. Scale bar = 50 μm. The arrows indicated the positive cells. (B) Representative images of mouse ESCs decidualization induced without (Control) or with 10 nM Estradiol (E_2_) and 1 μM Progesterone (P_4_) (d‐MESCs) for 5 days. The arrows indicated the typical cells. Scale bar = 100 μm. (C) PRL3 and PRL8 mRNA expression was determined by RT‐PCR in mouse ESCs subject to without or with 10 nM E_2_ and 1 μM P_4_. The graph represented the relative levels of mRNA expression in three separate experiments. The expression levels of mRNA were normalized against GAPDH expression. **p < 0.01, compared with control group. (D) CCK‐8 assay was performed to determine the viability of d‐MESCs treated with activin A or FST for 24 h. The absorbance was measured at 450 nm with a microplate spectrophotometer. Data represented mean ± SD (n = 3). *p < 0.05, compared with E_2_ + P_4_ group.

The cultured stromal cells from mouse endometrium were assessed by immunocytochemistry using the antibody against Vimentin (a marker of stromal cells).^26^ As shown in Figure 1A, cells isolated from endometrium were positive for anti‐Vimentin immunoreactivity, indicating that the cells belonged to stromal cells and could be used for subsequent experiments. Treatment with 10 nM E_2_ and 1 μM P_4_ induced a decidual cell phenotype characterized by cellular enlargement. Most decidual cells have a regular cobblestone shape, while some stromal cells also have jagged aspects (Figure 1B). The mRNA expression of prolactin (PRL), a decidualization marker in the endometrium, was detected by RT‐PCR. The transformation of decidual cells from ESCs was indicated by the expression of PRL3 and PRL8 mRNA (Figure 1C). To explore whether d‐MESCs belong to the target cells in response to FST or activin A, the cell viability was examined using a CCK‐8 kit. The results showed that 5 ng/ml activin A significantly promoted the viability of d‐MESCs, and 10 ng/ml FST slightly increased the viability of d‐MESCs (Figure 1D), indicating that FST and activin A can act on d‐MESCs. Thus, we performed all subsequent experiments using 5 ng/ml activin A and 10 ng/ml FST in d‐MESCs.

### 3.2 Effects of activin A and FST on adhesion and wound healing of d‐MESCs

**FIGURE 2:**
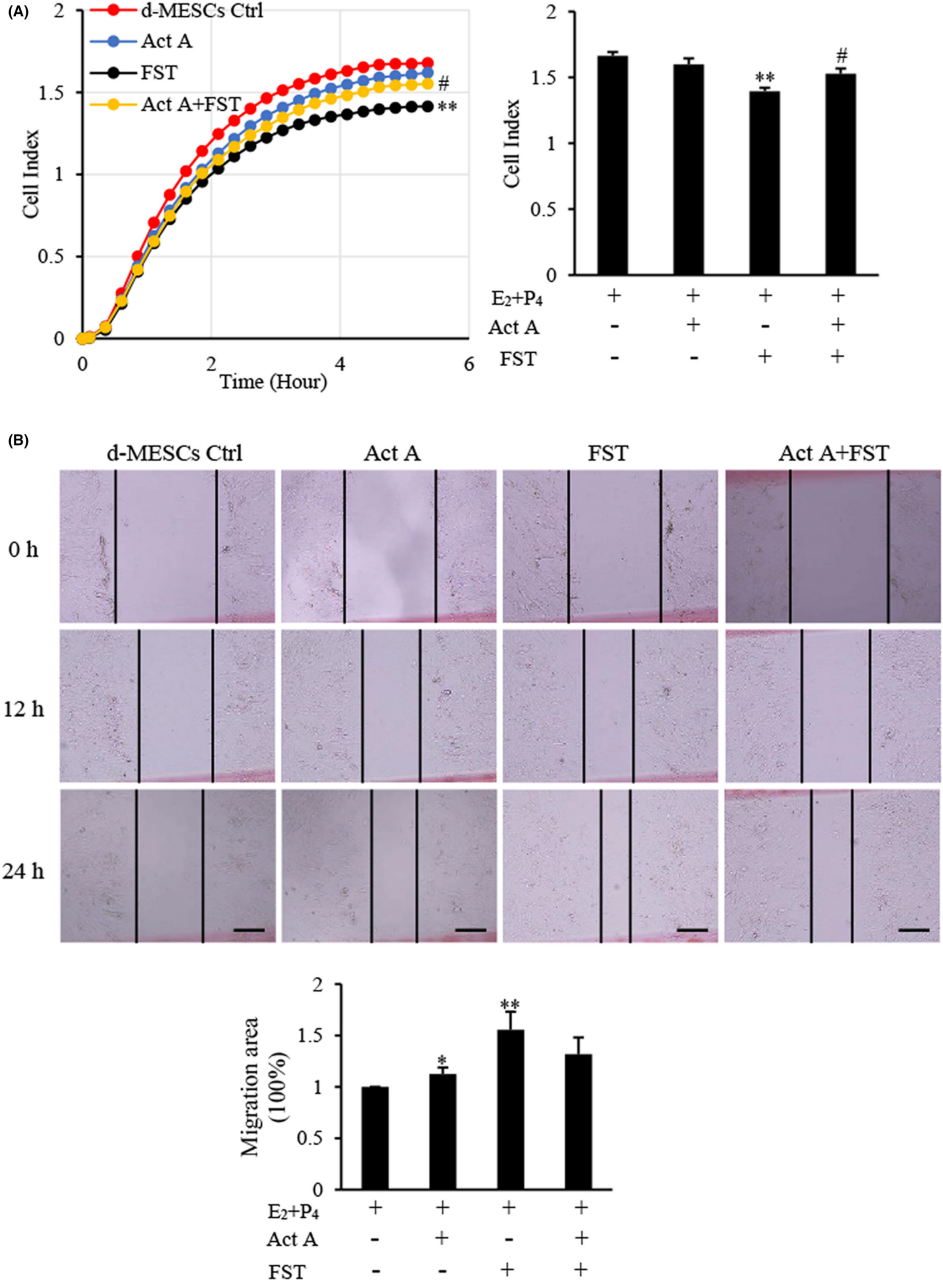
Effects of activin A and FST on adhesion and wound healing of d‐MESCs. (A) The adhesion was assessed by real‐time cell analysis (RTCA) in d‐MESCs subject to activin A 5 ng/ml or/with FST 10 ng/ml for 6 h. The graph showed Cell Index from three separate experiments. **p < 0.01, compared with d‐MESCs control group. #*p* < 0.05, compared with FST group. (B) A scratch‐wound was generated in monolayer d‐MESCs, and then, cells were treated with activin A 5 ng/ml or/and FST 10 ng/ml for 24 h. The graph showed the degree of wound healing from three separate experiments. Scale bar = 250 μm. *p < 0.05, **p < 0.01, compared with d‐MESCs control group.

To investigate the effects of activin A and FST on the biological behaviour of d‐MESCs, real‐time cell analysis (RTCA) was performed to examine cell adhesion.^29^ As shown in Figure 2A, activin A slightly decreased the adhesion of d‐MESCs, while FST inhibited the adhesion of d‐MESCs significantly, but FST action was attenuated by activin A. Scratch assays revealed that both activin A and FST promoted wound healing of d‐MESCs, while FST exerted more significant effect, but such promoting effects of FST were also attenuated by activin A (Figure 2B).

### 3.3 Effects of activin A and FST on migration of d‐MESCs

**FIGURE 3:**
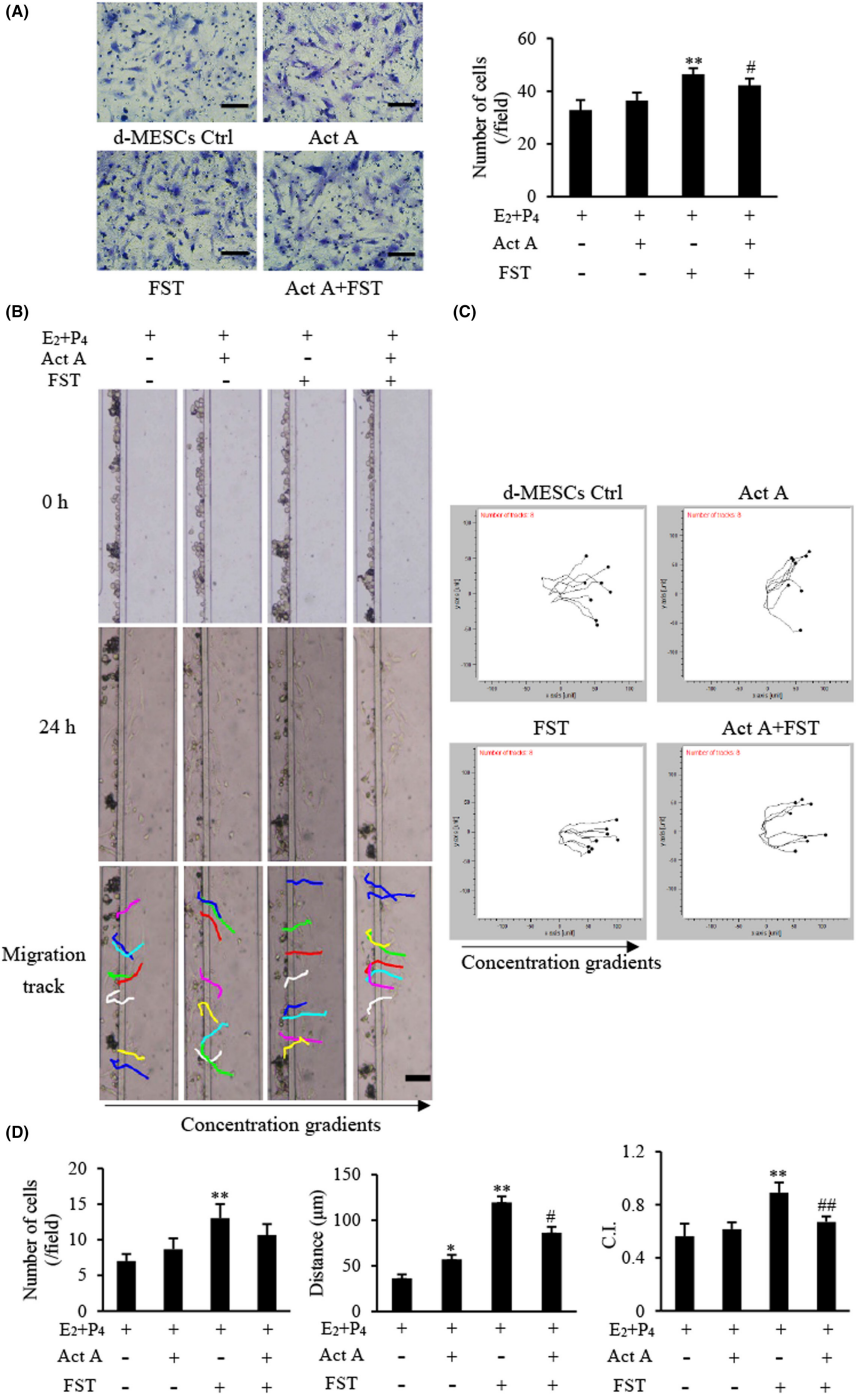
Effects of activin A and FST on migration of d‐MESCs. (A) The migration of d‐MESCs induced by activin A 5 ng/ml or/and FST 10 ng/mL was analysed by transwell migration assay. Cells that passed through porous membrane were stained with Giemsa. Scale bar = 100 μm. The graph showed the average number of migrated cells in three separate experiments. **p < 0.01, compared with d‐MESCs control group. #*p* < 0.05, compared with FST group. (B) Images of mouse d‐MESCs migration towards different concentrations activin A 5 ng/ml or/and FST 10 ng/ml were taken in the microfluidic device at 0 h and 24 h, respectively. Scale bar = 100 μm. (C) The tracked cell trajectories in activin A and/or FST gradient were analysed by Chemotaxis and Migration Tool software. Images represented the directions of migrated cell treated with activin A 5 ng/ml or/and FST 10 ng/ml. (D) The graph showed the average number, distance and chemotactic index (C.I.) of migrated cells in the same size fields of the microfluidic device in three separate experiments. *p < 0.05, **p < 0.01 compared with d‐MESCs control group. #*p* < 0.05, ##*p* < 0.01 compared with FST group.

In vivo, reduced cell adhesion and increased motility are both related to cell migration. Therefore, we tested the directional migration of d‐MESCs towards activin A and/or FST using a transwell assay. Among four groups, the total number of migratory cells was the largest in the FST group, followed by the FST combined with activin A group, while activin A alone did not increase the number of migratory cells (Figure 3A).

To better evaluate the cell migratory ability, such as distance, speed and direction of cell migration, we applied an established microfluidic platform to further examine the migration of d‐MESCs induced by activin A and FST. The results showed that FST significantly induced the migration of d‐MESCs, increasing not only the number of migratory cells but also the migration distance, while activin A only increased the migration distance of d‐MESCs significantly, but did not have a significant effect on the number of migrated cells (Figure 3B,D). The chemotactic index (C.I.) calculated from single cell tracking data further confirmed the directionality of d‐MESCs, and FST showed stronger directivity among four groups (Figure 3C,D). Similar to the results above, activin A suppressed the enhanced migratory capacity of d‐MESCs induced by FST.

### 3.4 Effects of activin A and FST on migration‐related proteins expression and calcium flux in d‐MESCs

**FIGURE 4:**
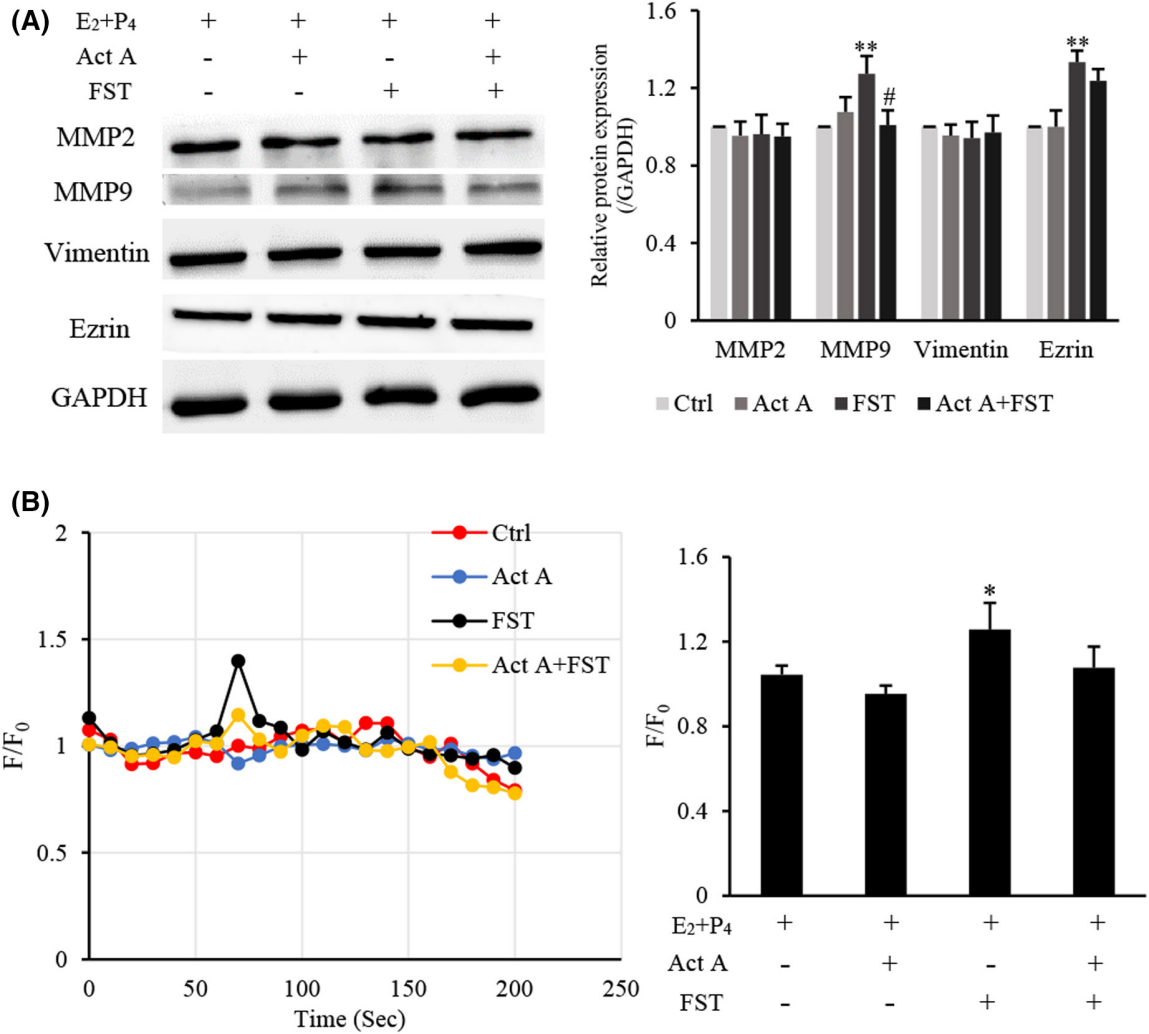
Effects of activin A and FST on migration‐related proteins expression and calcium flux in d‐MESCs. (A) Levels of MMP2, MMP9, Vimentin and Ezrin protein expressions were examined by Western blotting in d‐MESCs subject to 5 ng/ml activin A and/or 10 ng/ml FST for 24 h. The graph represented the relative levels of proteins in three separate experiments. The expression levels of these proteins were normalized against GAPDH, and the results were shown as the fold‐increase of the d‐MESCs control group. **p < 0.01, compared with d‐MESCs control group. #*p* < 0.05, compared with FST group. (B) Kinetics of calcium level was measured by the Fluo‐4 intensity normalized to the baseline (F/F_0_) in d‐MESCs treated with 5 ng/ml activin A or/and 10 ng/ml FST. The graph showed the peak values of calcium signal from three separate experiments. *p < 0.05, compared with d‐MESCs control group.

MMP2, MMP9 and ezrin are known to be involved in cell migration, and vimentin is an important marker for mesenchymal/motile phenotype.^30^, ^31^ Therefore, to determine the mechanism of action of activin A and FST on d‐MESCs migration, MMP2, MMP9, Vimentin and Ezrin expressions were analysed by Western blotting. The results showed that the expressions of MMP9 and Ezrin were upregulated significantly after FST stimulation, and only slightly increased after activin A treatment (Figure 4A). Neither activin A nor FST had a significant effect on MMP2 and Vimentin expression.

Calcium signalling also plays a vital role in cell proliferation, apoptosis and migration. In this study, the effects of activin A and FST on intracellular calcium in d‐MESCs were determined by flow cytometry. The results revealed that calcium levels were increased in response to FST treatment significantly (Figure 4B), while activin A antagonized partially FST action on calcium flux, suggesting that the effects of FST on d‐MESCs migration might be also associated with calcium signalling.

### 3.5 Effects of activin A and FST on expression of signalling proteins in d‐MESCs

**FIGURE 5:**
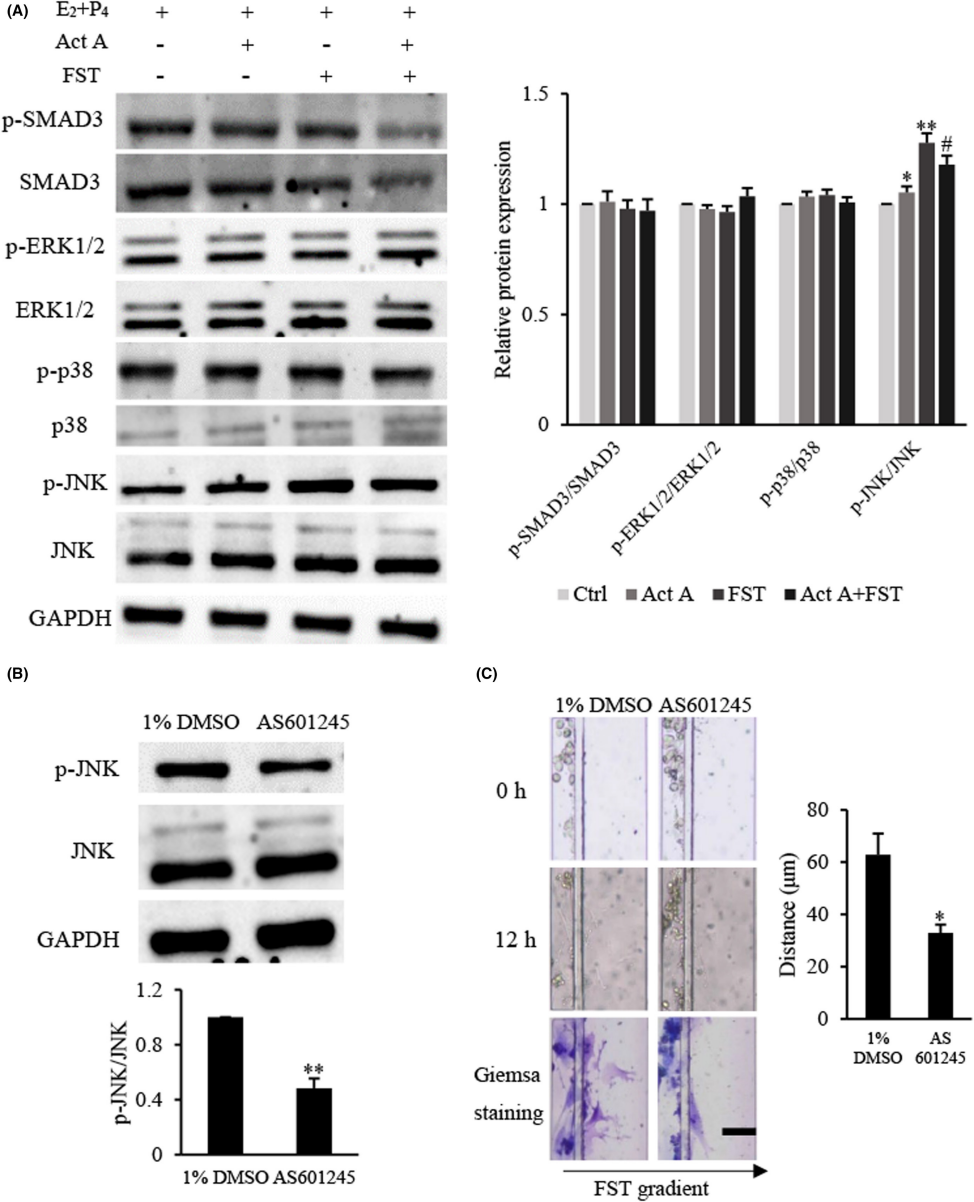
Effects of activin A and FST on expression of signalling proteins in d‐MESCs. (A) The levels of p‐SMAD3, SMAD3, p‐ERK1/2, ERK1/2, p‐p38, p38, p‐JNK and JNK proteins were examined by Western blotting in d‐MESCs subject to 5 ng/ml activin A or/and 10 ng/ml FST for 1 h. The graph represented the relative levels of protein in three separate experiments, and the results were shown as the fold‐increase of the d‐MESCs control group. *p < 0.05, **p < 0.01 compared with d‐MESCs control group. #*p* < 0.05, compared with FST group. (B) d‐MESCs were pretreated for 1 h with 1% DMSO or 1 μM JNK inhibitor AS601245; then, levels of p‐JNK protein were examined by Western blotting. The graph represented the relative levels of proteins in three separate experiments. **p < 0.01 compared with 1% DMSO d‐MESCs control group. (C) d‐MESCs were pretreated with 1% DMSO or 1 μM AS601245 for 1 h; cell migration towards 10 ng/ml FST gradient was examined by microfluidic device. *p < 0.05, compared with 1% DMSO d‐MESCs control group. Scale bar = 100 μm.

The above results showed that FST was a more effective chemoattractant regulating d‐MESCs migration; however, the signalling pathway is unclear. In this section, the protein expressions of canonical and non‐canonical activin A signalling pathways were examined by Western blotting (Figure 5A). The results revealed that activin A and FST had no significant effect on the levels of p‐SMAD3/SMAD3, indicating that activin A and FST might affect d‐MESCs activities through non‐canonical pathways. Furthermore, it was found that activin A and FST activated JNK, but not ERK1/2 and p38, resulting in significantly elevated levels of phosphorylation of JNK.

To determine whether FST‐induced JNK activation was responsible for d‐MESCs migration, we used JNK inhibitor AS601245 to repeat the migration assay. d‐MESCs were pretreated with 1% DMSO or 1 μM AS601245 diluted with 1% DMSO for 1 h. We found that AS601245 treatment decreased level of p‐JNK protein and reduced the migratory ability of d‐MESCs towards FST (Figure 5B,C). Taken together, the results indicated that FST might induce d‐MESCs migration through JNK signalling.

### 3.6 Effects of activin A and FST on viability in DSCs

**FIGURE 6:**
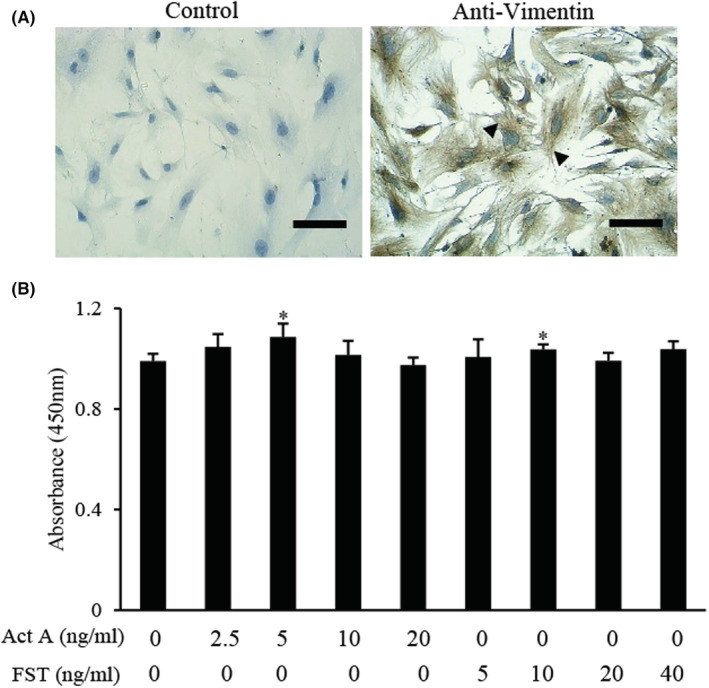
Effects of activin A and FST on viability of primary cultured decidual stromal cells (DSCs). (A) Immunocytochemical staining was performed to detect Vimentin expression in isolated mouse DSCs. Scale bar = 100 μm. The arrows indicated the positive cells. (B) CCK‐8 assay was performed to examine the viability of primary cultured DSCs treated with activin A or FST for 24 h. *p < 0.05, compared with control group.

To verify the above results, decidual stromal cells (DSCs) were isolated from the uterus of pregnant mouse. As shown in Figure 6A, immunocytochemical staining for anti‐Vimentin revealed that the isolated cells belonged to stromal cells and could be used for subsequent experiments. Moreover, the cell viability was measured using CCK‐8 assay after 24 h incubation and we found that 5 ng/ml activin A and 10 ng/ml FST promoted viability of primary cultured DSCs of pregnant mouse (Figure 6B).

### 3.7 Effects of activin A and FST on DSCs migration

**FIGURE 7:**
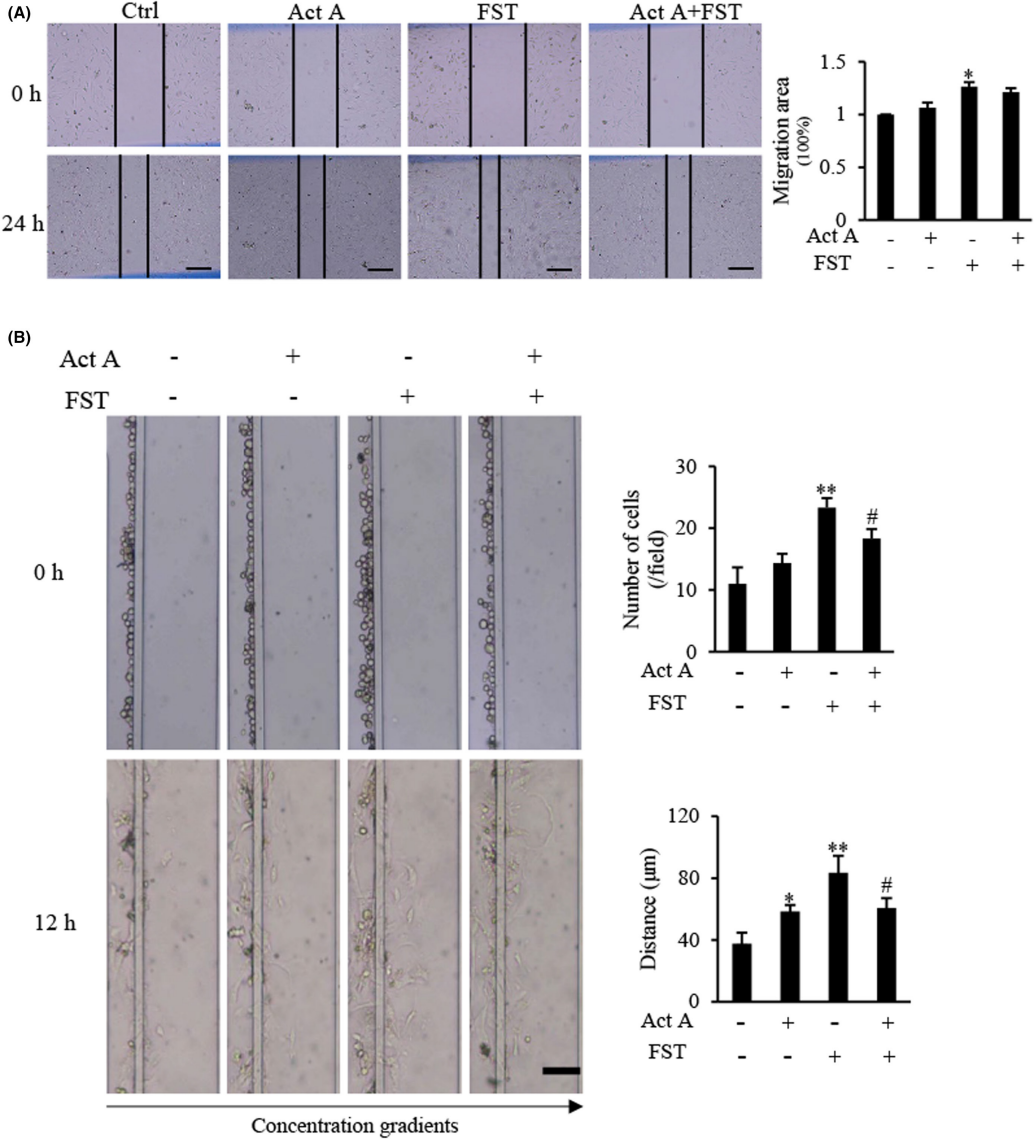
Effects of activin A and FST on migration of primary cultured DSCs from pregnant mouse. (A) A scratch‐wound was created in monolayer DSCs, and then, cells were treated with 5 ng/ml activin A or/and 10 ng/ml FST for 24 h. Scale bar = 250 μm. The graph showed the degree of wound healing from three separate experiments. *p < 0.05, compared with control group. (B) Images of mouse DSCs migration towards 5 ng/ml activin A or/and 10 ng/ml FST were taken in the microfluidic device at 0 h and 12 h, respectively. Scale bar = 100 μm. The graph showed the average number and distance of migrated cells in the same size fields of the microfluidic device in three separate experiments. *p < 0.05, **p < 0.01, compared with control group. #*p* < 0.05, compared with FST group.

Wound healing assay revealed that FST promoted wound healing of DSCs, while activin A exerted an antagonistic effect on FST (Figure 7A). Next, the migration of DSCs was determined by microfluidic devices. We found that the migratory number and distance of DSCs induced by FST increased significantly compared with control group. Although there was no significant difference in the number of migrated DSCs induced by activin A alone (*p* > 0.05), there was a considerable difference in the migration distance of DSCs, compared with the control group (*p* < 0.05) (Figure 7B). These data further confirmed that FST was a more effective chemoattractant for inducing the migration of d‐MESCs.

## 4 DISCUSSION

FST and activin A, as gonadal proteins, are involved in many physiological and pathological processes, such as inflammation, immunoregulation, angiogenesis and reproduction. Here, we analysed the effects of activin A and FST on the adhesion and migration of d‐MESCs by RTCA and microfluidic device. Firstly, ESCs were isolated and induced to decidualization in vitro. We found that FST promoted wound healing, inhibited adhesion and induced migration of d‐MESCs, while activin A neutralized the effects of FST on adhesion and migration of d‐MESCs. Next, primary cultured DSCs from uterus of pregnant mouse were used to further confirm the effects of activin A and FST, and the DSCs migration results were similar to d‐MESCs. These data suggest that compared with activin A, FST in activin A‐FST system is a more effective chemoattractant for inducing the migration of d‐MESCs.

The decidualization of ESCs is a prerequisite for embryo implantation. Under the stimulation of estradiol (E_2_) and progesterone (P_4_), ESCs undergo obviously morphological changes and possess the multinucleated appearance and are linked tightly.^32^ The functional layer of human endometrium changes in morphology and function every month and during pregnancy. From the middle secretory stage, human ESCs differentiate into decidual cells and secrete a variety of growth factors and cytokines. These molecules promote uterine decidualization and regulate the invasion of trophoblast cells. Decidualization initially occurs in ESCs around the spiral arteries and spreads to the whole endometrium once pregnancy occurs.^14^, ^33^ Vimentin as a marker of stromal cells is highly expressed in stromal cells no matter decidualization happens or not,^34^ and prolactin is widely used as a decidualization marker.^35^, ^36^

To investigate whether FST or activin A act on d‐MESCs, the cell viability was determined using a CCK‐8 kit. We found that activin A and FST both promoted DSCs viability, indicating that d‐MESCs are the target cells in response to FST or activin A. In this study, 5 ng/ml activin A and 10 ng/ml FST were selected as working concentration. Real‐time cell analysis (RTCA) is a technology based on the principle of microelectronic biosensor, which can realize the real‐time analysis of cells without markers in the process of experiment.^37^ In the present study, we found that FST inhibited the adhesion of d‐MESCs significantly, while activin A did not alter d‐MESCs adhesion but neutralized FST action. Moreover, the scratch wound experiments also showed that FST promoted wound healing of d‐MESCs, while activin A had an antagonistic effect on FST.

Cell migration involves the degradation of extracellular matrix, decreased cell adhesion and enhanced cell chemotaxis.^5^, ^38^ In this study, the migration of d‐MESCs was first tested by transwell migration assay. However, transwell assays are limited to only measure migrated cell numbers but lacking the ability to characterize quantitative cell motility and chemotaxis parameters at the single cell level such as cell migration speed, distance and directionality. In this regard, the microfluidic device offered quantitative insights into migratory responses of d‐MESCs in well‐controlled chemoattractant gradient conditions.^39^ We found that although activin A did not increase the number of migrated d‐MESCs, it extended the migration distance of d‐MESCs. FST not only induced a significant increase in the number of migrated d‐MESCs, but also significantly prolonged the migration distance of d‐MESCs, and with more obvious directionality. The primary cultured DSCs from uterus of pregnant mouse were used to further verify the above results, and results of primary cultured DSCs migration were similar to d‐MESCs. These findings indicate that compared with activin A, FST in activin A‐FST system is a more effective chemoattractant to induce the migration of d‐MESCs, which might be more conducive to uterine tissue remodelling during pregnancy.

A variety of functional proteins are involved in degradation of extracellular matrix, such as MMPs, ERM proteins (ezrin, radixin and moesin), PI3K/Akt and Ras‐mitogen‐activated protein kinases (MAPK).^40^, ^41^ MMPs are critical molecules of extracellular matrix remodelling and can promote cell motility through cytoskeletal rearrangement.^42^ Currently, MMPs consist of 23 members in human and are expressed in almost all organs and tissues, among them, MMP2 and MMP9 are the main markers of cell migration and invasion,^43^, ^44^ while MMP9 plays a more effective role in regulating cell invasion than MMP2, and MMP9 is the only one that gene deletion leads to the decline of fertility, suggesting that MMP9 plays an important role in the reproductive system.^45^ Worthy of note, ezrin is one of the numbers of ERM, which was first isolated from chicken intestinal brush borders. It is the connector between cortical actin filament and cell membrane and involved in the physiological processes such as microvilli formation, cell membrane structure change and cell adhesion. In cells over‐expressed ezrin, the migration ability is enhanced.^46^ Our data revealed that FST, but not activin A, promoted expression of MMP9 and Ezrin in d‐MESCs, suggesting that FST might induce d‐MESCs migration by up‐regulating Ezrin and MMP9.

In addition, cell migration is also related to many intracellular ions flux. Ca^(2+)^, an important second messenger in cells, regulates a variety of activities of cells, including cell migration, angiogenesis and inflammatory response.^47^ The increase of intracellular calcium flux leads to the activation of a variety of signal pathways, furthermore, the disintegration of intercellular adhesion and cytoskeleton rearrangement.^48^ In this study, we found that FST significantly enhanced the calcium signal of d‐MESCs, while activin A neutralized FST action on calcium flux. Previous study has also shown that activin A can promote the migration of L929 cells and breast cancer cells through calcium pathway.^28^, ^29^ Our data support the conjecture that FST might induce migration of d‐MESCs through increasing calcium influx.

Activin A binds to ActRII and activates shared canonical SMADs‐dependent signalling pathway. In addition, MAPK, PI3K/AKT, WNT and Notch are activated by activin A, which in turn can transduce the signalling of the independent SMAD proteins, and this cascade constitutes the non‐canonical pathways.^49^ As an activin binding protein, FST shows high affinity for activin and prevents activin from binding to its signalling receptor.^21^ However, FST receptor and its specific signalling pathway are still unclear. In the present study, we found no difference in the levels of p‐SMAD3/SMAD3, p‐ERK1/2/ERK1/2 and p‐p38/p38 in d‐MESCs, but obvious increase in the levels of p‐JNK/JNK. Moreover, JNK inhibitor AS601245 significantly attenuated FST action on inducing migration of d‐MESCs, suggesting that FST in activin A‐FST system, not activin A, is a crucial chemoattractant for inducing migration of d‐MESCs by JNK signalling.

The previous study has indicated that a conditional knockout of FST (FST‐cKO) results in a poor decidualization.^23^ Our data suggest that FST‐cKO may result in mDSCs migration disorder and failure of uterine remodelling to cause decidualization dysplasia. Activin A and FST are essential regulator for decidualization is undisputed; however, decidualization process is extremely complicated, also influenced by many other cells and molecules. Our findings provide a basis for preliminary experiments and possess reference significance for later research.

In summary, this study indicates that an important chemoattractant is FST rather than activin A to induce the migration of d‐MESCs by JNK signalling pathway. The maintenance of the balance of FST‐activin A system is very important for regulating remodelling of uterus during pregnancy. Our findings suggest that FST may be used as a treatment target and a potential indicator for predicting d‐MESCs migration and uterine decidualization, and administration of exogenous FST may improve decidua remodelling during pregnancy by inducing d‐MESCs migration, subsequently, increase the success rate of assisted reproductive technology.

### 4.1 Limitations

There are some limitations in our study. Firstly, these data have revealed that FST is an effective chemoattractant to induce mDSCs migration, but FST's specific receptor is still mysterious. Secondly, mouse decidualization in vitro was discussed, but there are still some differences in the pattern, biological characteristic with that in human. Human decidualization is a spontaneous process, while this study is induced in vitro by estradiol and progesterone. More studies should be carried out to explore the mechanism of the migration of decidualized human endometrial stromal cells and the relationship with pregnancy diseases.

## AUTHOR CONTRIBUTIONS

**Guole Liu:** Data curation (lead); formal analysis (lead); investigation (equal); methodology (equal); software (lead); validation (lead); visualization (lead); writing – original draft (lead). **Yan Qi:** Formal analysis (supporting); validation (supporting). **Jiandong Wu:** Writing – review and editing (equal). **Francis Lin:** Writing – review and editing (equal). **Xueling Cui:** Conceptualization (equal); investigation (equal); methodology (equal); project administration (equal); resources (equal); supervision (supporting); validation (supporting); writing – original draft (supporting). **Zhonghui Liu:** Conceptualization (equal); funding acquisition (lead); investigation (equal); methodology (equal); project administration (lead); resources (lead); supervision (lead); writing – original draft (equal).

## FUNDING INFORMATION

This research was funded by National Natural Science Foundation of China (31871510), the National Basic Research Program of China (2015CB943300), Natural Science Foundation of Jilin Province (20200201142JC) and Health Commission Foundation of Jilin Province (2020 J031 and 2019 J013).

## CONFLICT OF INTEREST

The authors declare no conflict of interest.

## Supporting information


Table S1

## Data Availability

The data that support the findings of this study are available from the corresponding author upon reasonable request.

## References

[1] Kajihara T , Tanaka K , Oguro T , et al. Androgens modulate the morphological characteristics of human endometrial stromal cells decidualized in vitro. Reprod Sci. 2014;21(3):372‐380. 23885104 10.1177/1933719113497280

[2] Li Q , Kannan A , Wang W , et al. Bone morphogenetic protein 2 functions via a conserved signaling pathway involving Wnt4 to regulate uterine decidualization in the mouse and the human. J Biol Chem. 2007;282(43):31725‐31732. 17711857 10.1074/jbc.M704723200

[3] Shao Q , Liu X , Huang Y , Chen X , Wang H . Human decidual stromal cells in early pregnancy induce functional Re‐programming of monocyte‐derived dendritic cells via crosstalk between G‐CSF and IL‐1β. Front Immunol. 2020;11:574270. 33193360 10.3389/fimmu.2020.574270 PMC7652738

[4] Ramathal CY , Bagchi IC , Taylor RN , Bagchi MK . Endometrial decidualization: of mice and men. Semin Reprod Med. 2010;28(1):17‐26. 20104425 10.1055/s-0029-1242989 PMC3095443

[5] Wu HM , Huang HY , Lee CL , Soong YK , Leung PC , Wang HS . Gonadotropin‐releasing hormone type II (GnRH‐II) agonist regulates the motility of human decidual endometrial stromal cells: possible effect on embryo implantation and pregnancy. Biol Reprod. 2015;92(4):98. 25761596 10.1095/biolreprod.114.127324

[6] Mathews LS . Activin receptors and cellular signaling by the receptor serine kinase family. Endocr Rev. 1994;15(3):310‐325. 8076584 10.1210/edrv-15-3-310

[7] Wijayarathna R , de Kretser DM . Activins in reproductive biology and beyond. Hum Reprod Update. 2016;22(3):342‐357. 26884470 10.1093/humupd/dmv058

[8] de Kretser DM , Hedger MP , Phillips DJ . Activin a and follistatin: their role in the acute phase reaction and inflammation. J Endocrinol. 1999;161(2):195‐198. 10320816 10.1677/joe.0.1610195

[9] Gao X , Zhao P , Hu J , et al. MicroRNA‐194 protects against chronic hepatitis B‐related liver damage by promoting hepatocyte growth via ACVR2B. J Cell Mol Med. 2018;22(9):4534‐4544. 30044042 10.1111/jcmm.13714 PMC6111826

[10] Massagué J , Chen YG . Controlling TGF‐beta signaling. Genes Dev. 2000;14(6):627‐644. 10733523

[11] Hedger MP , Winnall WR , Phillips DJ , de Kretser DM . The regulation and functions of activin and follistatin in inflammation and immunity. Vitam Horm. 2011;85:255‐297. 21353885 10.1016/B978-0-12-385961-7.00013-5

[12] Martinez PA , Li R , Ramanathan HN , et al. Smad2/3‐pathway ligand trap luspatercept enhances erythroid differentiation in murine β‐thalassaemia by increasing GATA‐1 availability. J Cell Mol Med. 2020;24(11):6162‐6177. 32351032 10.1111/jcmm.15243 PMC7294138

[13] Bloise E , Ciarmela P , Dela Cruz C , Luisi S , Petraglia F , Reis FM . Activin a in mammalian physiology. Physiol Rev. 2019;99(1):739‐780. 30540228 10.1152/physrev.00002.2018

[14] Jones RL , Salamonsen LA , Zhao YC , Ethier JF , Drummond AE , Findlay JK . Expression of activin receptors, follistatin and betaglycan by human endometrial stromal cells; consistent with a role for activins during decidualization. Mol Hum Reprod. 2002;8(4):363‐374. 11912285 10.1093/molehr/8.4.363

[15] Jones RL , Findlay JK , Farnworth PG , Robertson DM , Wallace E , Salamonsen LA . Activin a and inhibin a differentially regulate human uterine matrix metalloproteinases: potential interactions during decidualization and trophoblast invasion. Endocrinology. 2006;147(2):724‐732. 16282351 10.1210/en.2005-1183

[16] Jones RL , Salamonsen LA , Findlay JK . Activin A promotes human endometrial stromal cell decidualization in vitro. J Clin Endocrinol Metab. 2002;87(8):4001‐4004. 12161551 10.1210/jcem.87.8.8880

[17] Matzuk MM , Kumar TR , Vassalli A , et al. Functional analysis of activins during mammalian development. Nature. 1995;374(6520):354‐356. 7885473 10.1038/374354a0

[18] Mather JP , Woodruff TK , Krummen LA . Paracrine regulation of reproductive function by inhibin and activin. Proc Soc Exp Biol Med. 1992;201(1):1‐15. 1326766 10.3181/00379727-201-43473

[19] Yu J , Dolter KE . Production of activin a and its roles in inflammation and hematopoiesis. Cytokines Cell Mol Ther. 1997;3(3):169‐177. 9426975

[20] Munz B , Hübner G , Tretter Y , et al. A novel role of activin in inflammation and repair. J Endocrinol. 1999;161(2):187‐193. 10320815 10.1677/joe.0.1610187

[21] Shimonaka M , Inouye S , Shimasaki S , Ling N . Follistatin binds to both activin and inhibin through the common subunit. Endocrinology. 1991;128(6):3313‐3315. 2036994 10.1210/endo-128-6-3313

[22] Phillips DJ , de Kretser DM . Follistatin: a multifunctional regulatory protein. Front Neuroendocrinol. 1998;19(4):287‐322. 9799587 10.1006/frne.1998.0169

[23] Fullerton PT Jr , Monsivais D , Kommagani R , Matzuk MM . Follistatin is critical for mouse uterine receptivity and decidualization. Proc Natl Acad Sci U S A. 2017;114(24):e4772‐e4781. 28559342 10.1073/pnas.1620903114 PMC5474784

[24] Hu SJ , Ren G , Liu JL , et al. MicroRNA expression and regulation in mouse uterus during embryo implantation. J Biol Chem. 2008;283(34):23473‐23484. 18556655 10.1074/jbc.M800406200

[25] Zhang C , Yang C , Li N , et al. Elevated insulin levels compromise endometrial decidualization in mice with decrease in uterine apoptosis in early‐stage pregnancy. Arch Toxicol. 2019;93(12):3601‐3615. 31642978 10.1007/s00204-019-02601-8

[26] Tan Y , Li M , Cox S , et al. HB‐EGF directs stromal cell polyploidy and decidualization via cyclin D3 during implantation. Dev Biol. 2004;265(1):181‐195. 14697362 10.1016/j.ydbio.2003.09.019 PMC4277116

[27] Zhang XH , Liang X , Liang XH , et al. The mesenchymal‐epithelial transition during in vitro decidualization. Reprod Sci. 2013;20(4):354‐360. 23302397 10.1177/1933719112472738 PMC4077516

[28] Xie D , Liu Z , Wu J , et al. The effects of activin a on the migration of human breast cancer cells and neutrophils and their migratory interaction. Exp Cell Res. 2017;357(1):107‐115. 28479070 10.1016/j.yexcr.2017.05.003

[29] Jiang L , Qi Y , Kong X , et al. Activin a as a novel chemokine induces migration of L929 fibroblasts by ERK signaling in microfluidic devices. Front Cell Dev Biol. 2021;9:660316. 34095123 10.3389/fcell.2021.660316 PMC8175620

[30] Xu W , Song Y , Li K , Zhang B , Zhu X . Quercetin inhibits adenomyosis by attenuating cell proliferation, migration and invasion of ectopic endometrial stromal cells. Drug des Devel Ther. 2020;14:3815‐3826. 10.2147/DDDT.S265066 PMC7519414 33061289

[31] Owusu‐Akyaw A , Krishnamoorthy K , Goldsmith LT , Morelli SS . The role of mesenchymal‐epithelial transition in endometrial function. Hum Reprod Update. 2019;25(1):114‐133. 30407544 10.1093/humupd/dmy035

[32] Zhang FL , Huang YL , Zhou XY , Tang XL , Yang XJ . Telocytes enhanced in vitro decidualization and mesenchymal‐epithelial transition in endometrial stromal cells via Wnt/β‐catenin signaling pathway. Am J Transl Res. 2020;12(8):4384‐4396. 32913513 PMC7476159

[33] Bell SC . The insulin‐like growth factor binding proteins? The endometrium and decidua. Ann N Y Acad Sci. 1991;622:120‐137. 1712156 10.1111/j.1749-6632.1991.tb37856.x

[34] Logan PC , Ponnampalam AP , Rahnama F , Lobie PE , Mitchell MD . The effect of DNA methylation inhibitor 5‐Aza‐2′‐deoxycytidine on human endometrial stromal cells. Hum Reprod. 2010;25(11):2859‐2869. 20823114 10.1093/humrep/deq238

[35] Zhang X , Fu LJ , Liu XQ , et al. nm23 regulates decidualization through the PI3K‐Akt‐mTOR signaling pathways in mice and humans. Hum Reprod. 2016;31(10):2339‐2351. 27604954 10.1093/humrep/dew191

[36] Wang C , Zhao M , Zhang WQ , et al. Comparative analysis of mouse decidualization models at the molecular level. Genes (Basel). 2020;11(8):935. 32823685 10.3390/genes11080935 PMC7465532

[37] Hamidi H , Lilja J , Ivaska J . Using xCELLigence RTCA instrument to measure cell adhesion. Bio Protoc. 2017;7(24):e2646. 10.21769/BioProtoc.2646 PMC5777632 29367941

[38] Wu HM , Huang HY , Soong YK , Leung PCK , Wang HS . Kisspeptin regulation of human decidual stromal cells motility via FAK‐Src intracellular tyrosine kinases. Hum Reprod. 2019;34(7):1291‐1301. 31188433 10.1093/humrep/dez061 PMC6613344

[39] Abbas Y , Oefner CM , Polacheck WJ , et al. A microfluidics assay to study invasion of human placental trophoblast cells. J R Soc Interface. 2017;14(130):20170131. 28566515 10.1098/rsif.2017.0131 PMC5454302

[40] Krishnan K , Bruce B , Hewitt S , Thomas D , Khanna C , Helman LJ . Ezrin mediates growth and survival in Ewing's sarcoma through the AKT/mTOR, but not the MAPK, signaling pathway. Clin Exp Metastasis. 2006;23(3–4):227‐236. 17028919 10.1007/s10585-006-9033-y

[41] Maekawa M , Yamamoto T , Kohno M , Takeichi M , Nishida E . Requirement for ERK MAP kinase in mouse preimplantation development. Development. 2007;134(15):2751‐2759. 17611221 10.1242/dev.003756

[42] Sancéau J , Truchet S , Bauvois B . Matrix metalloproteinase‐9 silencing by RNA interference triggers the migratory‐adhesive switch in Ewing's sarcoma cells. J Biol Chem. 2003;278(38):36537‐36546. 12847101 10.1074/jbc.M304300200

[43] Murphy G , Nagase H . Progress in matrix metalloproteinase research. Mol Aspects Med. 2008;29(5):290‐308. 18619669 10.1016/j.mam.2008.05.002 PMC2810947

[44] Shi X , Luo X , Chen T , et al. Naringenin inhibits migration, invasion, induces apoptosis in human lung cancer cells and arrests tumour progression in vitro. J Cell Mol Med. 2021;25(5):2563‐2571. 33523599 10.1111/jcmm.16226 PMC7933922

[45] Li YF , Xu XB , Chen XH , Wei G , He B , Wang JD . The nuclear factor‐κB pathway is involved in matrix metalloproteinase‐9 expression in RU486‐induced endometrium breakdown in mice. Hum Reprod. 2012;27(7):2096‐2106. 22587999 10.1093/humrep/des110

[46] Tsukita S , Yonemura S . Cortical Actin organization: lessons from ERM (ezrin/radixin/moesin) proteins. J Biol Chem. 1999;274(49):34507‐34510. 10574907 10.1074/jbc.274.49.34507

[47] Dalal PJ , Muller WA , Sullivan DP . Endothelial cell calcium signaling during barrier function and inflammation. Am J Pathol. 2020;190(3):535‐542. 31866349 10.1016/j.ajpath.2019.11.004 PMC7074364

[48] Lange I , Koster J , Koomoa DT . Calcium signaling regulates fundamental processes involved in neuroblastoma progression. Cell Calcium. 2019;82:102052. 31306997 10.1016/j.ceca.2019.06.006

[49] Cui X , Shang S , Lv X , Zhao J , Qi Y , Liu Z . Perspectives of small molecule inhibitors of activin receptor‐like kinase in anti‐tumor treatment and stem cell differentiation (review). Mol Med Rep. 2019;19(6):5053‐5062. 31059090 10.3892/mmr.2019.10209 PMC6522871

