# Supplementary material for: Follistatin is a crucial chemoattractant for mouse decidualized endometrial stromal cell migration by JNK signalling
Source: J Cell Mol Med. 2022 Dec 18;27(1):127–40. doi: 10.1111/jcmm.17648 (PMC9806297; doi:10.1111/jcmm.17648)
Supplement: Supplementary file 1 — Table S1 [file JCMM-27-127-s001.docx]

Follistatin is a crucial chemoattractant for mouse decidualized endometrial stromal cell migration by JNK signaling

**Guole Liu^1^, Yan Qi^1^, Jiandong Wu^2^, Francis Lin^3^, Zhonghui Liu^1*^ and Xueling Cui^4*^**

^1^ Department of Immunology, College of Basic Medical Sciences, Jilin University, Changchun 130021, China

^2^ Institute of Biomedical and Health Engineering, Shenzhen Institute of Advanced Technology, Chinese Academy of Sciences, Shenzhen 518055, China

^3^ Department of Physics and Astronomy, University of Manitoba, Winnipeg, MB, R3T 2N2, Canada

^4^ Department of Genetics, College of Basic Medical Sciences, Jilin University, Changchun 130021, China

*Correspondence: [liuzh@jlu.edu.cn (Z.L.)](mailto:liuzh@jlu.edu.cn%20(Z.L.)) and [cxl@jlu.edu.cn](mailto:cxl@jlu.edu.cn) (X.C.); Tel.:86-431-8561-9476 (Z.L.) and +86-431-8561-9487 (X.C.)

Table S1

Table S1. Primer sequences for RT-PCR.

| Gene | Primer Sequence | Accession number | | Fragment size (bp) |
| --- | --- | --- | --- | --- |
| PRL3 | GCCACACGATATGACCGGAA | [NM_001163218.1](https://www.ncbi.nlm.nih.gov/entrez/viewer.fcgi?db=nucleotide&id=253735809) | | 162 |
|  | GGTTTGGCACATCTTGGTGTT |  | |  |
| PRL8 | AGCCAGAAATCACTGCCACTC | [NM_001289919.1](https://www.ncbi.nlm.nih.gov/entrez/viewer.fcgi?db=nucleotide&id=583966121) | | 119 |
|  | TGATCCATGCACCCATAAAA |  | |  |
| GAPDH | GATTGTTGCCATCAACGACC | [NM_001289726.1](https://www.ncbi.nlm.nih.gov/entrez/viewer.fcgi?db=nucleotide&id=576080554) | | 372 |
|  | GTGCAGGATGCATTGCTGAC | |  |  |
